# Supplementary material for: Reversible Thermo-Optical Response Nanocomposites Based on RAFT Symmetric Triblock Copolymers (ABA) of Acrylamide and N-Isopropylacrylamide and Gold Nanoparticles
Source: Polymers (Basel). 2023 Apr 21;15(8):1963. doi: 10.3390/polym15081963 (PMC10144036; doi:10.3390/polym15081963)
Supplement: Supplementary file 1 [file polymers-15-01963-s001.zip › polymers-2287506-supplementary.pdf]

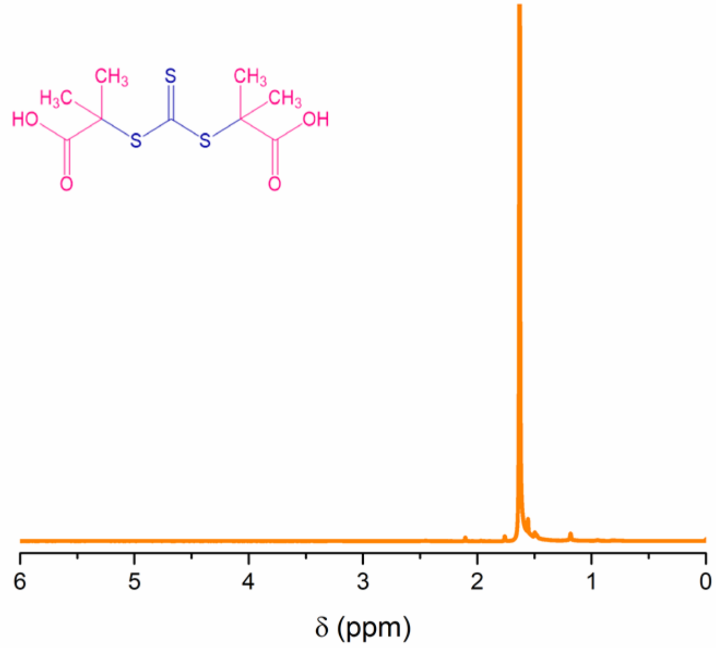

Figure S1. ^{1}H NMR spectrum of the symmetric transfer agent 2,2'-(thiocarbonylbis(sulfanediyl))bis(2-methylpropanoic acid).

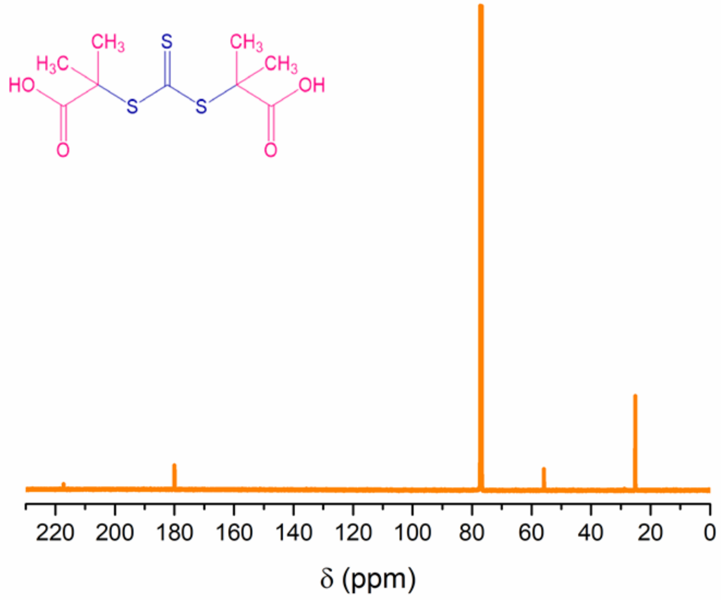

Figure S2. ^{13}C NMR spectrum of the symmetric transfer agent 2,2'-(thiocarbonylbis(sulfanediyl))bis(2-methylpropanoic acid).

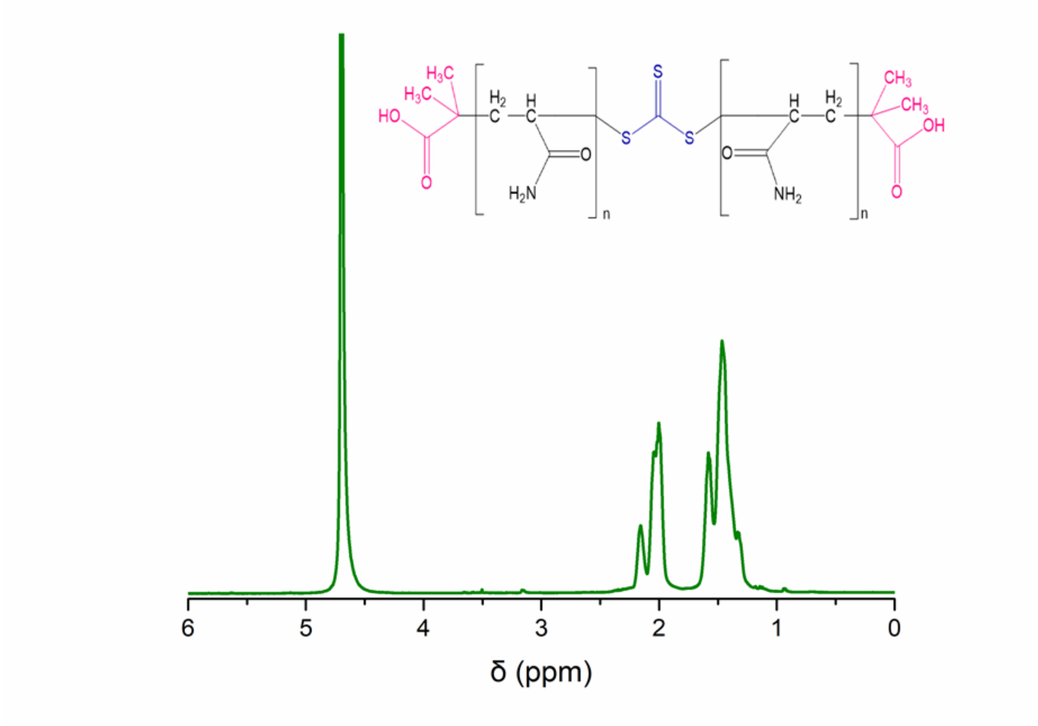

Figure S3. ^{1}H NMR spectrum of the polyacrylamide block (PAM).

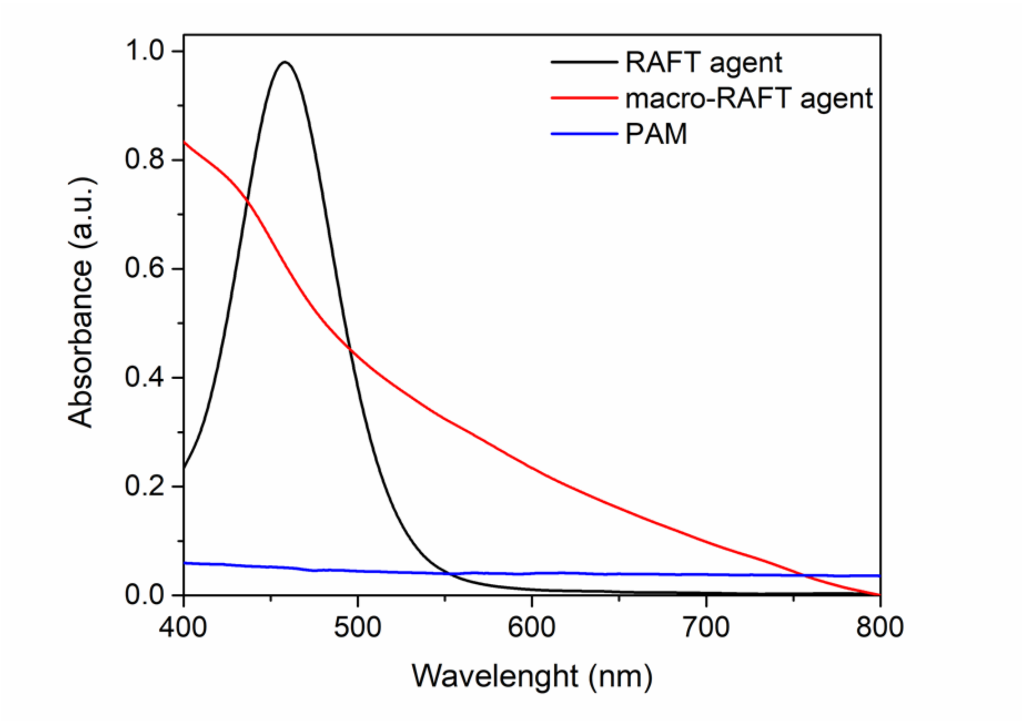

Figure S4. Comparison of UV-Vis spectra of symmetric transfer agent 2,2'-(thiocarbonylbis(sulfanediyl))bis(2-methylpropanoic acid), the macro-RAFT agent (the PAM block) and polyacrylamide (PAM) synthesized by inverse emulsion polymerization.

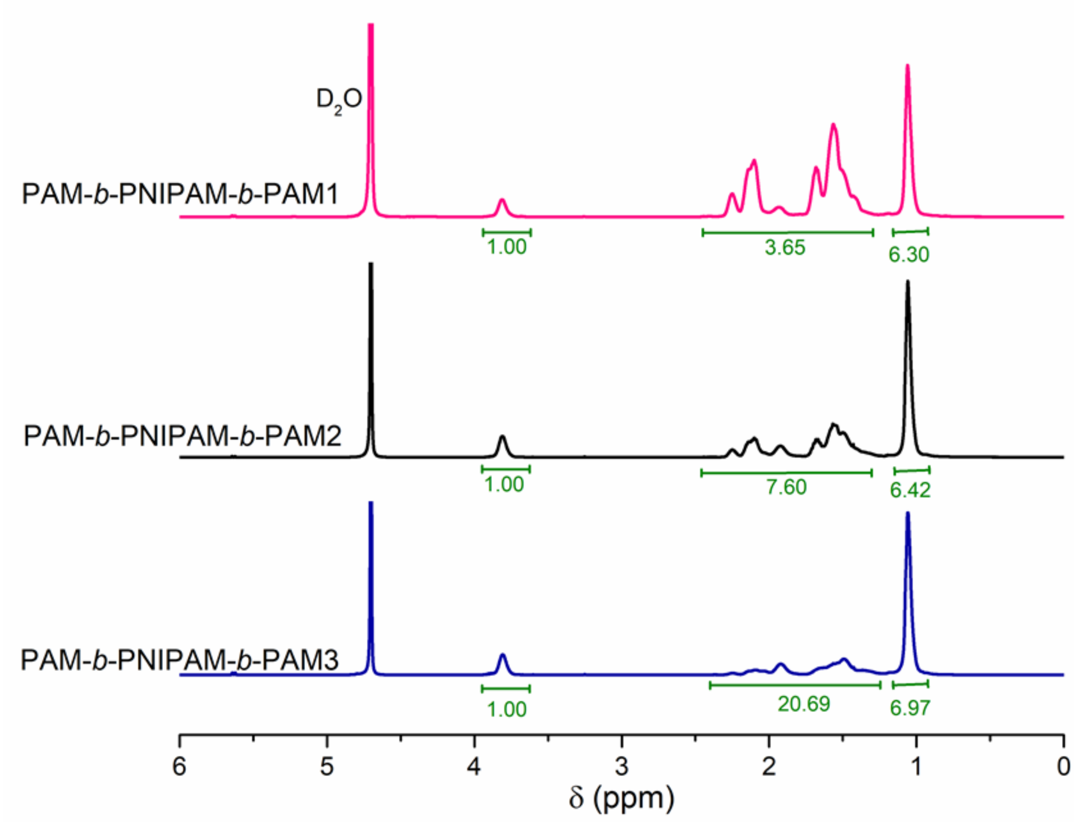

Figure S5.^{1}H NMR spectrum, with the signal integration of PAM-*b*-PNIPAM-*b*-PAM1, PAM-*b*-PNIPAM-*b*-PAM2 and PAM-*b*-PNIPAM-*b*-PAM3.
